# Changes to cholesterol trafficking in macrophages by *Leishmania* parasites infection

**DOI:** 10.1002/mbo3.469

**Published:** 2017-03-27

**Authors:** Geo Semini, Daniel Paape, Athina Paterou, Juliane Schroeder, Martin Barrios‐Llerena, Toni Aebischer

**Affiliations:** ^1^ Mycotic and Parasitic Agents and Mycobacteria Department of Infectious Diseases Robert Koch‐Institute Berlin Germany; ^2^ Institute of Immunology and Infection Research The University of Edinburgh Edinburgh UK; ^3^ Present address: Welcome Trust Centre for Molecular Parasitology and Institute of Infection Immunity and Inflammation College of Medical, Veterinary and Life Sciences, University of Glasgow Glasgow UK; ^4^ Present address: Centre for Cardiovascular Sciences Queen's Medical Research Institute University of Edinburgh Edinburgh UK

**Keywords:** cholesterol, filipin staining, *Leishmania*, macrophages, Niemann–Pick C1 (NPC1), parasitophorous vacuole

## Abstract

*Leishmania* spp. are protozoan parasites that are transmitted by sandfly vectors during blood sucking to vertebrate hosts and cause a spectrum of diseases called leishmaniases. It has been demonstrated that host cholesterol plays an important role during *Leishmania* infection. Nevertheless, little is known about the intracellular distribution of this lipid early after internalization of the parasite. Here, pulse‐chase experiments with radiolabeled cholesteryl esterified to fatty acids bound to low‐density lipoproteins indicated that retention of this source of cholesterol is increased in parasite‐containing subcellular fractions, while uptake is unaffected. This is correlated with a reduction or absence of detectable NPC1 (Niemann–Pick disease, type C1), a protein responsible for cholesterol efflux from endocytic compartments, in the *Leishmania mexicana* habitat and infected cells. Filipin staining revealed a halo around parasites within parasitophorous vacuoles (PV) likely representing free cholesterol accumulation. Labeling of host cell membranous cholesterol by fluorescent cholesterol species before infection revealed that this pool is also trafficked to the PV but becomes incorporated into the parasites’ membranes and seems not to contribute to the halo detected by filipin. This cholesterol sequestration happened early after infection and was functionally significant as it correlated with the upregulation of mRNA‐encoding proteins required for cholesterol biosynthesis. Thus, sequestration of cholesterol by *Leishmania* amastigotes early after infection provides a basis to understand perturbation of cholesterol‐dependent processes in macrophages that were shown previously by others to be necessary for their proper function in innate and adaptive immune responses.

## 1 Introduction

*Leishmania* spp. are trypanosomatid parasites that are transmitted as flagellated extracellular metacyclic promastigotes into the skin of the mammalian host by the bite of a female sandfly. After uptake by host phagocytes, promastigotes differentiate to nonflagellated intracellular amastigotes. Amastigotes represent the parasites forms that persist in the infected host and are responsible for the infection of other cells recruited to the lesions, thereby inducing the propagation of the disease (Kima, 2007). In the host cell, similar to *Coxiella burnetii* but in contrast to mycobacteria or *Salmonella*,* Leishmania* amastigotes thrive in a habitat designated as parasitophorous vacuole (PV) that is thought to resemble a late endosome/early lysosome based on the presence of marker proteins characteristic of these compartments such as Rab7 and LAMP‐1/‐2, respectively (Antoine, Prina, Jouanne, & Bongrand, 1990; Kima et al., 2010; Russell, Xu, & Chakraborty, 1992). We and others have shown that maturation kinetics of parasite‐infested phagosomes follow a uniform maturation sequence compatible with that of the general endocytic pathway (Collins, Schaible, Ernst, & Russell, 1997; Duclos et al., 2000; Lippuner et al., 2009).

Previous proteome analysis of intracellular *Leishmania* parasites performed in our laboratory (Paape, Barrios‐Llerena, Le Bihan, Mackay, & Aebischer, 2010; Paape et al., 2008) confirmed a drastic difference in metabolism between amastigotes and promastigotes (Hart & Coombs, 1982). A switch toward fatty acid catabolism in amastigotes has also been reported for *Leishmania donovani* (Rosenzweig, Smith, Myler, Olafson, & Zilberstein, 2008). In addition, this outcome has been corroborated by studies using isotope‐resolved metabolomics, where amastigotes exhibited a glucose‐sparing metabolism associated with increased rates of beta‐oxidation (Saunders et al., 2014). Thus, these data together with the rising significance of endocytically low‐density lipoprotein (LDL)‐derived cholesterol during parasitic infections (Bansal, Bhatti, & Sehgal, 2005; Hamid et al., 2015; Johndrow, Nelson, Tanowitz, Weiss, & Nagajyothi, 2014) prompted our interest in lipid trafficking and metabolism in the host cell, and in the *Leishmania*‐containing PV in particular.

Several recent investigations already recognized the importance of cholesterol during *Leishmania* infection. It has been demonstrated that parasite phagocytosis is dependent on normal plasma membrane cholesterol levels (Chattopadhyay & Jafurulla, 2011; Pucadyil, Tewary, Madhubala, & Chattopadhyay, 2004; Tewary, Veena, Pucadyil, Chattopadhyay, & Madhubala, 2006). Moreover, through a yet to be defined mechanism, infection leads to reduced levels of this lipid in the plasma membrane thereby affecting what are considered lipid raft‐dependent processes (Chakraborty et al., 2005; Sen, Roy, Mukherjee, Mukhopadhyay, & Roy, 2011). Transcriptomic studies of the host cells showed that *Leishmania* infection modulated the expression of genes associated with cholesterol biosynthesis, uptake, and efflux (Osorio y Fortea et al., 2009; Rabhi et al., 2012). Finally, mouse models (Fernandes et al., 2013; Ghosh, Bose, Roy, & Bhattacharyya, 2013; Ghosh et al., 2012; Shamshiev et al., 2007) and epidemiological data (Ghosh et al., 2011; Lal et al., 2007; Soares et al., 2010) suggest a correlation between hypocholesterolemia and hypolipidemia and progression of the disease. However, the kinetics and mechanism (s) leading to alterations in cholesterol distribution in host cell membranes, its extent, and effect on its biosynthesis are still not understood.

This study reports new insight into the potential cause of the abovementioned findings by establishing that infection with *Leishmania* is associated with an increased retention of exogenous cholesterol and fatty acids bound to LDL in the PV. This correlates with a concentration of sequestered free cellular cholesterol around the parasite, detectable as a halo. The extent of sequestration is dependent on the number of intracellular parasites. Furthermore, host cell membrane‐bound cholesterol, while not contributing to the cholesterol halo, becomes quickly incorporated into the parasites. Finally, this two‐way cholesterol sequestration and incorporation by the parasites amounts to one third of the host cell whole cholesterol and is associated with transcriptional upregulation of host cell genes that are involved in cholesterol biosynthesis and are induced by the ER resident, cholesterol‐level‐sensitive transcription factor SREBP (sterol regulatory element‐binding protein).

## 2 Experimental procedures

### 2.1 Growth and differentiation of *L*. *mexicana*

A clone of *L*. *mexicana mexicana* (MNYC/BZ/62/M379) expressing *DsRed* (Sorensen et al., 2003) and *L*. *major* LRC‐L137 V121 wild type (Misslitz, Mottram, Overath, & Aebischer, 2000) were cultured in semidefined culture medium (SDM) supplemented with 10% heat‐inactivated fetal bovine serum, 0.1 mmol/L adenine, 1 μg ml^−1^ biotin, 5 μg ml^−1^ hemin, and 2 μg ml^−1^ biopterin (all from Sigma‐Aldrich). Differentiation of *L*. *mexicana* promastigotes to amastigotes in axenic culture was carried out as described previously (Paape et al., 2008).

### 2.2 Ethics statement

All animal experiments adhered to the UK Animals (Scientific Procedures) Act 1986 and were approved by Project License No. 6003581 granted by the Home Office (U.K.) to T.A. and conducted in accordance with local guidelines.

### 2.3 Infection of bone marrow‐derived macrophages

Bone marrow was flushed from femura of CBA/J mice purchased from Harlan UK (Loughborough, UK) and maintained in a conventional animal facility. Macrophages were differentiated from bone marrow of 6–8 weeks old female mice as described previously (Weinheber, Wolfram, Harbecke, & Aebischer, 1998), and infected with axenic *L*. *Mexicana::DsRed* amastigotes or *L. major* promastigotes at a multiplicity of infection of 5 or 10, respectively. Two hours after addition, excess parasites were washed out and macrophages were further incubated at 33°C and 5% CO_2_ before harvesting.

### 2.4 Low‐density lipoprotein labeling

For labeling of LDL, the lipid dispersion technique was done according to Groener, Pelton, & Kostner (1986). Briefly, 5 μCi of cholesteryl linoleate [cholesteryl‐1 2 6 7‐3H (N)] (American Radiolabeled Chemicals, St. Louis, USA) was mixed with 1 μmol phosphatidylcholine (Sigma‐Aldrich, St. Louis, USA) and 20 nmol of butylated hydroxytoluene (Sigma‐Aldrich, St. Louis, USA) in chloroform. Chloroform was evaporated in a stream of nitrogen. Ester and phosphatidylcholine were dissolved in 50 mmol/L Tris HC1, pH 7.4, containing 0.1 g L^−1^ EDTA and the tube was flushed with nitrogen. The suspension was sonicated twice (RK100H Sonorex, Bandelin, Berlin, Germany) for 5 min at RT. Sonicated mixture was added to 5 mg LDL (Sigma‐Aldrich, St. Louis, USA) and 5 ml lipoprotein‐deficient serum (LPDS; Sigma‐Aldrich, St. Louis, USA), 0.6 ml of a 10 mmol L^−1^ 5,5‐dithiobis(2‐nitrobenzoic acid) (Sigma‐Aldrich, St. Louis, USA) solution, and 80 μl of a 100 g L^−1^ EDTA solution were added. The tube was flushed with nitrogen and incubated for 24 hr at 37°C.

After labeling, the LDL was obtained by density‐gradient ultracentrifugation as described by Redgrave et al. (1975). Briefly, the density of mixture was adjusted with KBr (Sigma‐Aldrich, St. Louis, USA) to 1.063 g ml^−1^ and centrifuged for 24 hr at ~286,000*g* at 4°C. LDL was floating on top of gradient and transferred into 1 ml of LPDS. To stabilize the LDL, porcine albumin was added to obtain a final concentration of 80 g L^−1^.

Macrophages were infected with *L*. *mexicana* amastigotes (MOI of 5) or beads (MOI of 5). Macrophages were maintained in DMEM/10% FCS/4% L929 conditioned medium. Medium was changed every 24 hr. Ninety‐six hours postinfection, infected as well as uninfected cells were incubated for 1 hr in medium containing 10% LDL‐depleted plasma and ^3^H‐labeled cholesteryl linoleate. Aliquots were taken before and after incubation. Labeled cholesterol containing medium was removed and cells were washed with normal medium and incubated for 2.5 hr at 34°C. An aliquot was withdrawn after the 2.5 hr incubation and cells were washed in 4°C DMEM medium. Next, cells were lysed in DMEM/0.008% SDS, transferred into a 1.5‐ml tube, and centrifuged at 1100*g*. The supernatant was retained and the pellet was washed three times with cold PBS. Subsequently aliquots of all samples except for the pellet fraction were applied onto a filter mat in duplicate. The mat was dried and a wax scintillant was melted onto the filter mat and subsequently evaluated in a scintillation counter (Wallac MicroBeta TriLux, PerkinElmer, Waltham, MA, USA). Data were analyzed using a general linear model which was conducted in Minitab version 15. Residual were log_10_ transformed and they showed homogeneity of variance. *p*s < .05 were considered statistically significant.

### 2.5 Fluorescence microscopy

Cultured macrophages were attached on 13‐mm diameter glass coverslips with 2 × 10^5^ cells. Macrophages were then infected either with axenic *L. mexicana* amastigotes, *L*. *major* promastigotes, or IgG‐coated 3‐μm latex beads (Polysciences, Eppelheim, Germany) at a multiplicity of infection of 5 and 10 (for promastigotes). In the case of coinfection of macrophages with parasites and latex beads ratio of one or five bead(s) per macrophage was used as indicated in the figure legends. Infected or bead exposed macrophages were incubated for 2 hr at 33°C and 5% CO_2_. For kinetic studies, cells were washed with PBS and fixed at the indicated time points postinfection.

Immunostaining of NPC‐1 and LAMP‐1 was carried out as follows: cells were fixed with 4% (v/v) PFA for 30 min, permeabilized with 0.5% saponin in buffer A (2% FCS, 0.1% BSA, 0.1% NaN_3_ in PBS) for 30 min, blocked in 3% BSA/PBS, and incubated for 1 hr with the appropriate primary antibody. NPC‐1 was detected using a primary polyclonal rabbit anti‐mouse NPC‐1‐antibody (AbCam, Cambridge, UK) and a secondary Cy5^®^ goat anti‐rabbit IgG. LAMP‐1 was detected using an Alexafluor 488‐conjugated rat anti‐mouse LAMP‐1 antibody, clone 1D4B (BD Biosciences, San Jose, USA). Primary and secondary antibodies were diluted in 0.5% saponin in buffer A. For cholesterol staining we used the polyene filipin. Cells were washed three times with PBS and fixed for 30 min at RT with 4% paraformaldehyde. After fixation, cells were washed with PBS and incubated with 1.5 g glycine in PBS 10 min at RT. Staining was performed with PBS/10% FCS containing 0.05 mg ml^−1^ filipin (25 mg ml^−1^ in DMSO, Sigma‐Aldrich, St. Louis, USA) for 1 hr at RT. After staining, cells were washed three times with PBS and the cover slips were mounted in Vectashield HardSet Mounting Medium (Vector Laboratories, Burlingame, USA).

For labeling of host cell cholesterol pool by fluorescent cholesterol species, macrophages were cultured as indicated above. Sixteen hours prior to infection, macrophages were incubated with 22‐NBD‐cholesterol (2.5 μmol/L, Life Technologies, Darmstadt, Germany) or TopFluor (BODIPY)‐cholesterol (0.5 μmol/L, Avanti Polar Lipids, Inc., Alabaster, USA), washed with PBS, and infected with *L*. *mexicana* amastigotes for the indicated time points. Then, cells were treated as described for filipin staining.

Cells were examined with a Leica TCS SP5 or Zeiss LSM 780 confocal laser scanning microscope with Axio Observer Z1 (Carl Zeiss, Jena, Germany). Images were acquired and analyzed with ZEN (Black Edition; Carl Zeiss, Jena, Germany) or Volocity 3D (Improvision/PerkinElmer) software. Filipin images were recorded with an AxioCam MRm camera (Carl Zeiss, Jena, Germany), 63× magnification, oil immersion. Filipin was excited with a 405‐nm laser, and fluorescence was detected in an emission window at 400–480 nm. The single optical section was estimated to be about 0.5‐μm thick. Processing, linescans, and cross‐section visualization of 2D and 3D images was performed with ZEN Lite 2012 Blue and Black Edition (Carl Zeiss, Jena, Germany). Quantification of the filipin intensity and NBD cholesterol fluorescence was performed by ImageJ (version 1.47m).

### 2.6 Purification of RNA and generation of complementary DNA

RNA was purified from 1.1×10^6^ *L*. *mexicana* amastigotes infected (MOI 5) or uninfected macrophages at 0, 24, 48, 72, and 96 hr postinfection. Infections were routinely done in six‐well tissue culture plates. Cells were washed three times with PBS, and 1 ml TRIzol (Invitrogen, Paisley, UK) was added to the well. Purification of RNA was performed according to the manufacturers’ instructions.

RNA was resuspended in a small volume of 17 μl of RNase‐free water. The purified RNA was treated with DNA‐free™ Kit (Ambion—Applied Biosystems, Invitrogen, Paisley, UK) according to the manufacturer's instructions. The RNA yield was determined with NanoDrop 1000 (Thermo Scientific, Waltham, USA).

The RNA was transcribed with ThermoScript^™^ RT‐PCR System (Invitrogen, Paisley, UK) according to the manufacturers’ instructions. Briefly, samples from all the time points were transcribed at once. Samples were adjusted with water to the respective amount of 9 μl RNA solution of sample with lowest RNA concentration. As primer oligo (dT)_20_ was used. Transcription was performed for 60 min at 50°C and the reaction products were treated with RNase H. The resulting cDNA was diluted with sterile water to the appropriate concentration.

### 2.7 Quantitative reverse transcription polymerase chain reaction

Reactions were performed in fast optical 96‐well plates (Applied Biosystems, Invitrogen, Paisley, UK). Reaction mixture was as follows: 10 μl of diluted cDNA, 5 μl primer mix (2 μl of each primer, 10 mmol/L), and 15 μl Power SYBR Green PCR Master Mix (Applied Biosystems, Invitrogen, Paisley, UK). Plates were sealed with MicroAmp Optical Adhesive Film (Applied Biosystems, Invitrogen, Paisley, UK). Quantitative reverse transcription polymerase chain reaction (qRT‐PCR) was performed with StepOnePlus real‐time PCR machine (Life Technologies, Carlsbad, USA). The amplification program was as follows: 50°C for 2 min, 95°C for 10 min, 40 cycles of 95°C for 15 s followed by 60°C for 1 min. After 40 cycles, a dissociation protocol was performed. Data were analyzed with StepOne software version 2.0. Primers used: *Fdps* F, GGGATGCTATTGCCCGGCTCAA; *Fdps* R GCTTCCAGAAGCAGAGCGTCGT; *Hmgcr* F, AATGTTGTCAAGACTTTTCCGGA; *Hmgcr* R, GTACTTGGACCCAAGCTGCCGTA; *Ldlr* F, GTGTGATGGAGACCGAGATT; *Ldlr* R, CTGCGATGGATACACTCACT; *Scd1* F, CGCCCAAGCTGGAGTACGTCTG; *Scd1* R, CACAAGCAGCCAACCCACGTG; *Sqle* F, CATCGTGGGATCTGGTGTGCTTGG; *Sqle* R, GCCATAGCTGCTTTCCGGAGACTC; *Hprt* F1, GGACCTCTCGAAGTGTTGGA; *Mhprt* R2, GGCCACAGGACTAGAACACC; *Hprt* F2, TGCTGACCTGCTGGATTACA; *Hprt2*, TCCAACACTTCGAGAGGTCC.

Upregulation or downregulation of genes of interest was determined by the comparative cycle threshold (C_T_) method (Livak & Schmittgen, 2001). Genes were normalized to the average C_T_ value of hypoxanthine–guanine phosphoribosyl transferase 1 (*Hprt*) obtained using the primer pairs hprt1 F/R and hprt2 F/R. In order to determine the relative change in gene expression upon infection, C_T_ values of genes of interest on infected cells were compared to uninfected cells at the respective time point. Prior to this, the C_T_ values of both the infected and uninfected cells were normalized to an endogenous housekeeping gene, hypoxanthine–guanine phosphoribosyl tranferase 1 (*hprt*). *Hprt* was chosen, because a study investigated the variation of 13 different housekeeping genes in five different types of normal and tumor tissue and revealed that *hprt* was the most accurate single normalization gene (de Kok et al., 2005). Not all samples could be analyzed in one run qRT‐PCR, therefore, to normalize between different runs of one experiment, an inter‐run calibrator was used. For this purpose, throughout all runs of one experiment, the same sample (0 hr uninfected) was used as template as well as the same primer mixtures for housekeeping genes (*hprt* and γ*‐actin*). To keep variation between time point low, for each target gene, all samples (uninfected and infected as well as all time points) were analyzed in the same run. Additionally, for *hprt* two primer pairs were used and the average between the C_T_ values of each pair was used for normalization. This was done to compensate for different amplification efficiencies of each primer pair. The amplification efficiency of all primer pairs was determined by generating standard curves. Primers were used when amplification efficiency was between 1.96 and 2.17, otherwise new sets were designed and ordered. Furthermore, the comparative C_T_ method assumes an amplification efficiency of 100% which corresponds to 2 and is also the base for the exponential amplification ($2^{-\Delta \Delta C_{T}}$). Here, due to only minor variations in the amplification efficiencies, the base for the exponential amplification was left at 2.

### 2.8 Statistical analyses

Data obtained from quantification analyses using ImageJ were subjected to Shapiro–Wilk normality test. If data were normally distributed, one‐way analysis of variance (ANOVA) was performed and datasets were compared using Bonferroni's multiple comparison test. If data were not normally distributed, nonparametric Kruskal–Wallis statistics were performed and datasets were compared using Dunn's multiple comparison test. A nonparametric Mann–Whitney *U* test was used to determine significant differences between linescans’ intensities for *L*. *mexicana* and latex beads.

## 3 Results

### 3.1 Uptake of cholesterol linoleate by *L*. *mexicana*‐infected macrophages

To investigate the extracellular cholesterol trafficking to the PV of infected macrophages, we employed a biochemical approach. Human as well as murine cells in peripheral organs are supplied with cholesterol and fatty acids bound to LDL. After endocytosis in a receptor‐mediated manner, LDL disintegrates in lysosomal compartments where cholesteryl esters are hydrolyzed to free fatty acids and free cholesterol. In LDL, cholesterol comprises 60% of the total lipid bound and approximately 80% of it is esterified with mainly unsaturated fatty acids (Goldstein & Brown, 1977). Using LDL containing ^3^H‐labeled cholesterol in the form of cholesteryl linoleate, we investigated cholesterol uptake by macrophages in pulse‐chase experiments. Cells were infected with amastigotes or exposed to latex beads and then pulsed with medium containing hot cholesteryl linoleate complexed to LDL. After 2½ hours, cells were lysed and large particles were centrifuged to collect the phagosomes that contained either latex beads or parasites. The radioactivity of the supernatant and the pellet was determined separately and total cell‐associated radioactivity was calculated and correlated with particle pellet retained activity. In the pellet fraction of uninfected macrophages, we measured only background‐level radioactivity, hence no cholesterol was sequestered in pelleted material of uninfected cells. Macrophages infected with amastigotes or exposed to latex beads exhibited proportions of retained cholesterol in the pellet fraction of 32% and 13%, respectively. Thus, the amastigote‐containing fraction of infected macrophages retained more than twice the amount of cholesterol than bead‐containing phagosomes, and this difference was statistically significant (Table 1, *p* = .011).

**Table 1:** Uptake and retention of cholesterol in uninfected amastigotes or beads exposed macrophages

	Uninfected macrophages	Macrophages infected with amastigotes (MOI 5)	Macrophages exposed to beads (5 beads per cell)
Activity associated with total cells (cpm)	1,135 ± 195	1,199 ± 32	1,570 ± 325
Activity retained by particle pellet (cpm)	33 ± 18	386 ± 81	209 ± 55
Proportion of retained cholesterol in pellet	0.03 ± 0.01	0.32 ± 0.07^a^	0.13 ± 0.03

a Significant difference, *p* = .011, in cholesterol retention in compartments harboring amastigotes compared to beads, 96 hr postinfection (analyzed with general linear model, *F*_1,4_ = 20.13; *n* = 3).

### 3.2 Localization of Niemann–Pick C1 (NPC1) protein in *L*. *mexicana*‐infected macrophages

**Figure 1:**
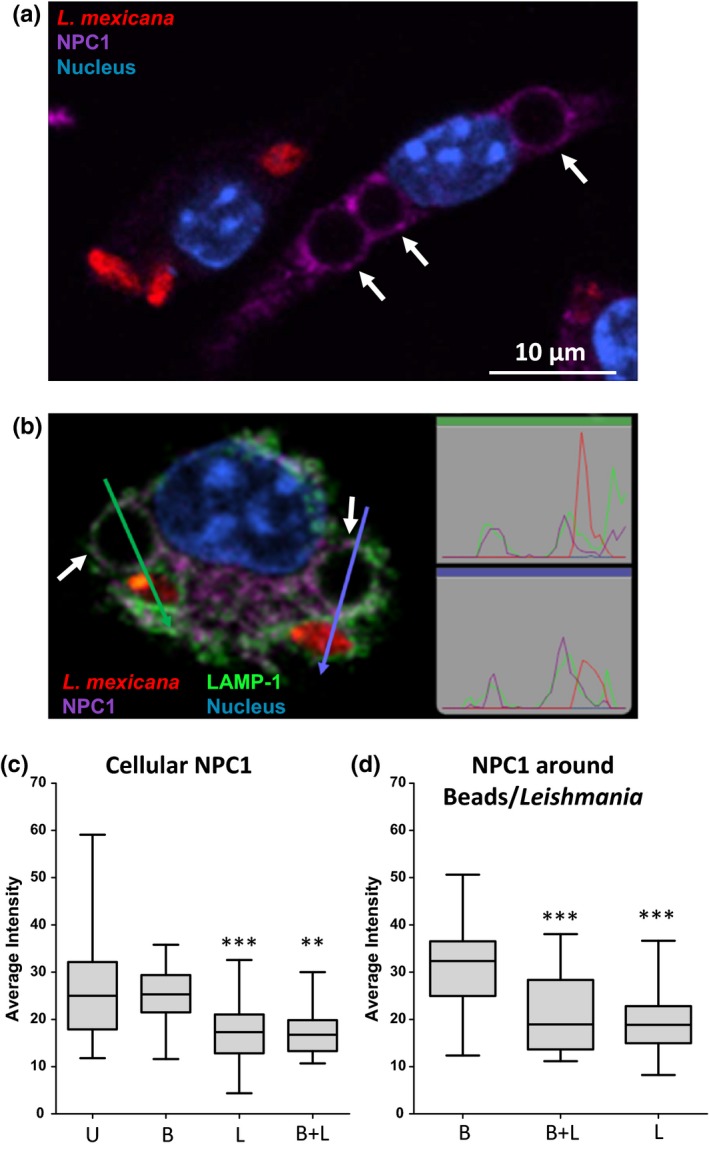
Niemann–Pick C1 (NPC1) accumulation is reduced in *Leishmania mexicana‐*infected macrophages. Macrophages were simultaneously infected with axenic *L*. *mexicana* amastigotes expressing *DsRed* (MOI of 3) and exposed to IgG‐coated latex beads (MOI of 3). Three hours after infection, macrophages were fixed, blocked, and incubated with antibodies recognizing NPC1 and LAMP‐1 (lysosomal membrane protein used as a marker of PVs). Nuclei were stained with DAPI. (a) Confocal image of two representative macrophages, which either exclusively contain *L. mexicana* amastigotes or latex beads. (b) Confocal image of one representative cell containing both *L. mexicana* amastigotes and latex beads. Arrows and charts outlined with the respective color refer to linescans (measured with Volocity 3D software) of adjacent parasite and latex beads, in order to highlight colocalization of NPC1 and LAMP‐1. (c) Quantification of the cytosolic NPC1 signal in macrophages using ImageJ software. The mean NPC1 signal intensity of the whole macrophage was normalized removing the unspecific NPC1 signal intensity and area associated with nucleus, parasite body, and latex bead. Four groups were defined: uninfected macrophages (U; *n* = 32), macrophages containing latex beads only (B; *n* = 28), macrophages containing parasites only (L; *n* = 44), and macrophages containing both parasites and beads (B + L; *n* = 16). Uninfected macrophages were set as control group for statistical analysis (***p < .001, **p < .01). (d) Quantification of the NPC1 signal in the outer ring of parasites and latex beads. The mean NPC1 signal intensity of the area that immediately surrounds latex beads and parasites with a thickness of 0.25 μm was calculated using ImageJ software. Three groups were defined: intensity ring of latex beads from macrophages without *Leishmania* parasites (B; *n* = 32), intensity ring of latex beads from macrophages containing parasites (B + L; *n* = 20), and intensity ring of parasites (L; *n* = 46). Data for B + L and L were compared with B (***p < .001). All images were acquired using Leica TCS SP5 microscope with a 100× oil‐immersion objective

Cholesterol levels in macrophages are normally regulated by cholesterol uptake, biosynthesis and efflux. Tubulovesicular trafficking of cholesterol out of the late endosome/early lysosome to the plasma membrane and/or to the endoplasmic reticulum is mediated at least by two proteins: Niemann–Pick C1 (NPC1) and NPC2 (Blanchette‐Mackie, 2000; Neufeld et al., 1999). It has been proposed that soluble NPC2 accepts cholesterol deriving from LDL and transports it to membrane‐bound NPC1 for export (Infante et al., 2008; Kwon et al., 2009). Consequently, mutations in either NPC1 or NPC2 are responsible for an accumulation of unesterified free cholesterol in late endosomal/lysosomal compartments (Carstea et al., 1997; Naureckiene et al., 2000). We investigated whether cholesterol retention in PVs may to a certain extent related to presence or absence of NPC1 protein. Analyses using immunofluorescent staining and confocal laser scanning microscopy (CLSM) demonstrated that NPC1 protein levels in bone marrow‐derived macrophages infected with *L*. *mexicana* were reduced, while the protein was easily detected at latex bead‐containing phagosomes (Figure 1a). Representative linescans of macrophages that enclosed both parasites and latex beads displayed a decreased NPC1 signal associated with the PVs compared to the NPC1 positive beads‐containing phagosomes (Figure 1b). Quantitative analysis showed that *L*. *mexicana*, in contrast to the latex beads, significantly diminished the cellular content of NPC1 to about 35%, independently of the presence of latex beads in the same cell (Figure 1c). In addition, similar results were also observed when only the region that immediately surrounded parasites and/or latex beads was analyzed. In cells containing parasites or beads, intensity of the NPC1 signal in the area around the parasites was 37% lower compared to the same area around the latex beads (Figure 1d). In cells containing parasites and beads, *L. mexicana*‐dependent NPC1 depletion was notable mostly around both phagosome types (Figure 1d); in certain cells, affecting more notably parasite‐containing phagosomes (Figure 1b).

### 3.3 Fate of cellular free cholesterol in the presence of *Leishmania* parasites

**Figure 2:**
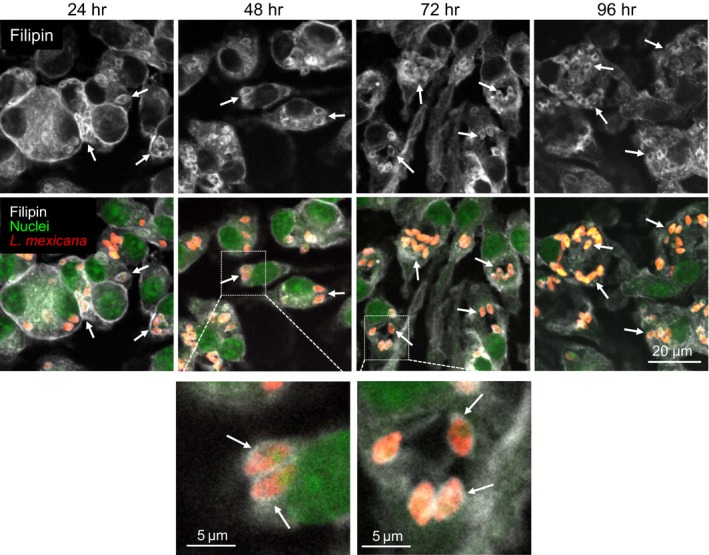
Filipin accumulation in macrophages upon *Leishmania mexicana* amastigotes infection. Macrophages were infected with *L. mexicana* amastigotes expressing DsRed (MOI of 5) for 2 hr. Distribution of filipin was analyzed over a time course of 96 hr, with time points every 24 hr. Cells were fixed and subsequently stained with filipin (white, excited at 405 nm) and SYTO ^®^ 13 (nuclei, green, excited at 488 nm). Arrows indicate the presence of *Leishmania* parasites (red) and/or filipin around the parasites. One representative of three independent experiments is shown. All images are confocal and were acquired using a Zeiss LSM 780 microscope, 63× oil‐immersion objective, and processed using identical conditions

**Figure 3:**
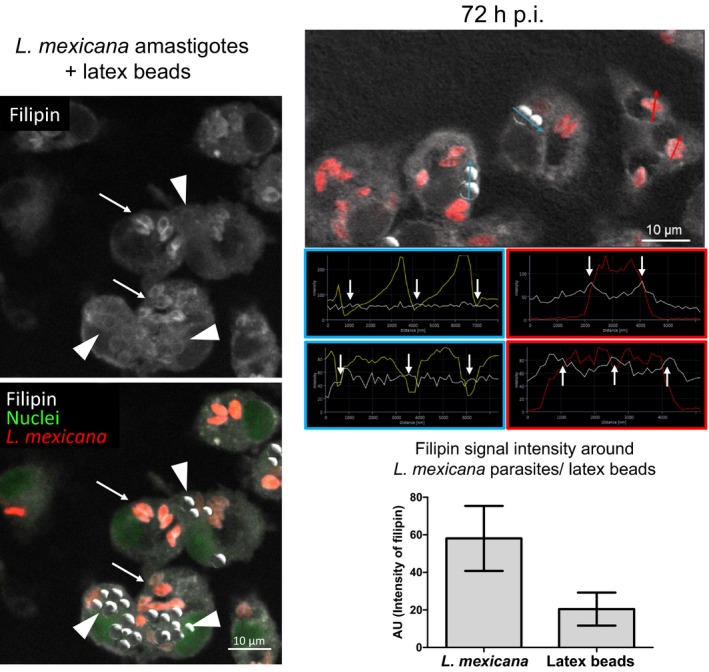
Distribution and quantification of the filipin signal around *Leishmania mexicana* amastigotes and latex beads. Macrophages were simultaneously infected with *L*. *mexicana* amastigotes (MOI of 5) and incubated with latex beads (five per cell) for 2 hr. Distribution of filipin was analyzed 72 hr after infection. Cells were fixed and subsequently stained with filipin (white, excited at 405 nm) and SYTO ^®^ 13 (nuclei, green, excited at 488 nm). Latex beads are visualized using the transmission channel. The image represents a confocal section acquired using a Zeiss LSM 780 microscope, 63× oil‐immersion objective. (left panel) Arrows indicate the presence *Leishmania* parasites (red, excited at 543 nm) and/or filipin‐stainable free cholesterol around the parasites. Arrowheads indicate selected latex beads incorporated by macrophages. One representative of three independent experiments is shown. (right panel top) Red arrows and charts outlined in red refer to representative linescans (measured with ZEN software) of *L*. *mexicana* parasites phagocytosed by macrophage. Arrows and charts outlined in blue refer to representative linescans of latex beads incorporated by macrophages. White arrows indicate signal intensities for filipin (white line) around latex beads (yellow line) and parasites (red line) that were used to quantify intensity differences in filipin staining. (right panel bottom) For quantification of filipin signal around internalized *L*. *mexicana* amastigotes and latex beads, maximal signal intensity (indicated as mean peak value in arbitrary units (AU) ± SD,* n* = 36 along section paths) was determined using ZEN (blue edition) software. Background values were subtracted from the absolute values. Differences between parasites and latex beads were highly significant *p* < .0001

**Figure 4:**
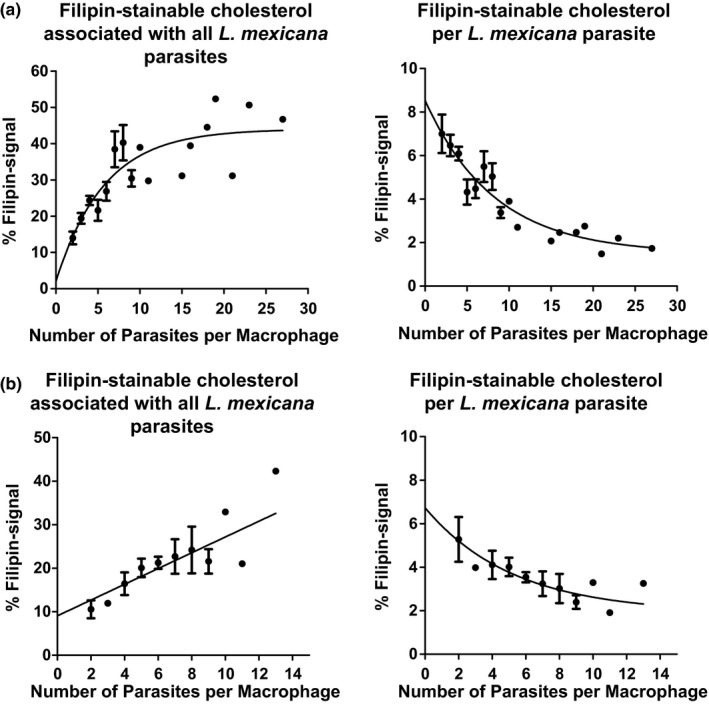
Amount of sequestered filipin per parasite is a function of the number of parasites per cell. Relative filipin signal of (a) confocal 2D sections and (b) 3D confocal microscope image stack processed in a 2D projection by maximum intensity projection is represented as a function of the number of parasites internalized by the macrophages over a period of 96 hr postinfection. The exponential curve regressions were calculated using GraphPad Prism

**Figure 5:**
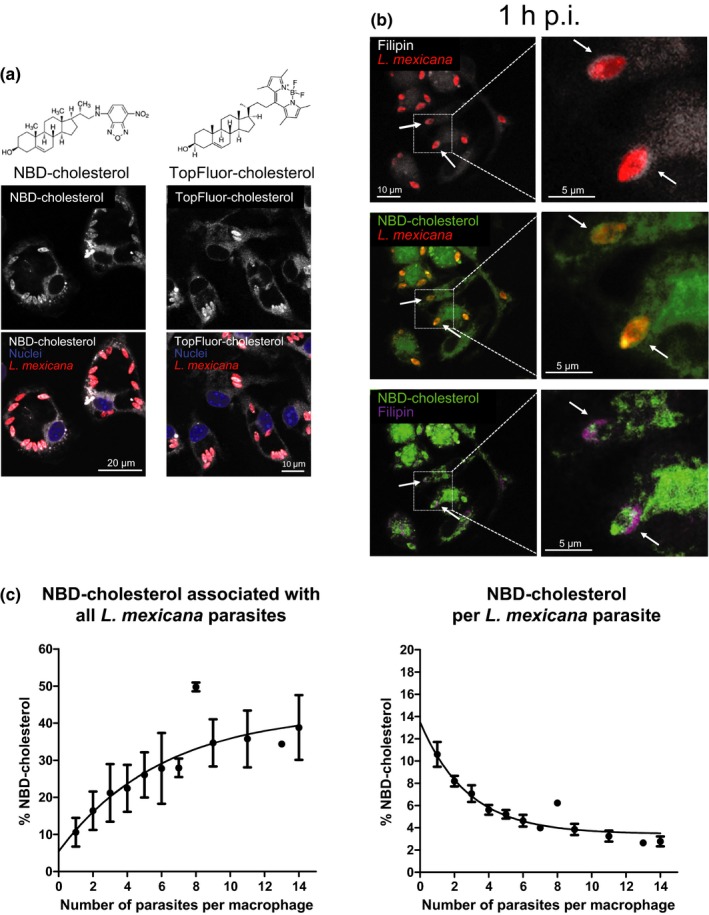
Fluorophore‐labeled cholesterol does not accumulate around the parasite. (a) Macrophages were preincubated with NBD‐ or TopFluor‐cholesterol for 16 hr. Afterward, macrophages were infected with *Leishmania mexicana* amastigotes (MOI of 5) for 2 hr. Seventy‐two hours after infection, cells were fixed and subsequently stained with DAPI (nuclei, excited at 405 nm). Both NBD and TopFluor groups were excited at 488 nm. (b) Distribution of filipin‐stainable and fluorophore‐labeled cholesterol in the early phases of infection with *L*. *mexicana*. Mouse bone marrow‐derived macrophages were preincubated with NBD‐cholesterol for 16 hr and then infected with *L*. *mexicana* amastigotes (MOI of 5) for 2 hr. An hour after infection, cells were fixed and subsequently stained with filipin (excited at 405 nm). For a better identification, filipin‐stained cholesterol in the presence of NBD‐cholesterol is visualized in purple. Arrows indicate *Leishmania* parasites and/or filipin around the parasites. All images are confocal and were acquired using a Zeiss LSM 780 microscope, 63× oil‐immersion objective, and processed using identical conditions. (c) Relative fluorescence corresponding to NBD‐cholesterol incorporated by all the parasites (left panel) or a single parasite (right panel) in an infected macrophage is represented as a function of the number of parasites internalized by the macrophages over a period of 48 hr postinfection. The exponential curve regressions were calculated using GraphPad Prism

As mentioned in the Introduction, previous studies indicate that *Leishmania* uptake by macrophages as well as infection maintenance is dependent on serum and cellular cholesterol. Moreover, it has been described that infection leads to depletion of cholesterol from the host cell plasma membrane, disturbing lipid raft‐dependent processes (Chakraborty et al., 2005; Rabhi et al., 2012). Nevertheless, little is known about the sequence of events, distribution, and extent and kinetics of changes in free cholesterol that might occur within the host cell in the course of *Leishmania* infection. For cholesterol staining, we used the polyene filipin. Although this antibiotic can also bind other β‐hydroxysterols and lipids (e.g., GM1, sphingomyelin, phosphatidylcholine), it is—because of the scarcity of reagents available—commonly used to visualize free cholesterol distribution in cells. Using this reagent, we first investigated the distribution of free cholesterol using CLSM. Image analysis revealed that filipin staining accumulated around *L. mexicana* amastigotes within the PV of infected macrophages. This accumulation of likely free cholesterol was detectable as a halo throughout a time course of 96 hr (Figure 2). Moreover, filipin signal accumulated already 1 hr after infection suggesting that this process begins very early during the infection (see further down and Figure5 . The filipin halo was also detected in PVs of macrophages infected with *L. major* promastigotes under the same experimental conditions (Figure S1), indicating that this event occurs with species that live in communal (*L*. *mexicana*) as well as individual PVs (*L. major*). In contrast, macrophages incubated with latex beads did not show an augmented filipin accumulation around the latex beads in their phagosomes (Figure S2). This result is further supported in experiments with macrophages that were simultaneously infected with *L*. *mexicana* amastigotes and incubated with latex beads (Figure 3a). Filipin staining of axenically grown amastigotes and promastigotes alone did not exhibit the characteristic cholesterol halo around the parasite, indicating that this process occurred specifically in PVs (data not shown). We also determined the extent of the filipin signal around both, internalized parasites and latex beads. Representative linescans displayed an increased filipin signal around the *L*. *mexicana* parasites, but not around latex beads (Figure 3b). Relative signal intensities were then quantified revealing a threefold enhanced accumulation of filipin‐stainable cholesterol around *Leishmania* parasites compared to latex beads (Figure 3c). Signal accumulation around internalized *L*. *mexicana* 24 hr after infection was further analyzed by a 3D visualization of a series of images using a surface reconstruction mode (Figure S3A) and by a cross‐sectional profile of a *z*‐stack (Figure S3B), which indicated a coat‐like distribution detected as a halo in 2D projections. Similar results were also obtained in *L*. *mexicana*‐infected macrophages 48, 72, and 96 hr postinfection (Figure S4–S6, respectively). In addition, 2D sections were quantified measuring the intensity (referred to as integrated density) of filipin‐stainable cholesterol around the parasites as well as incorporated by host cells. This analysis showed that about 15% and 44% of the host cell filipin signal becomes associated with the parasites in infected macrophages with low and high pathogen load, respectively (Figure 4a). Regression analysis of these data suggested that the maximal amount of signal sequestered had a limit and consequently the amount per parasite was a function of the number of parasites per cell, and was not time dependent (data not shown). 3D confocal microscope image stacks, which were then processed for 2D representation by maximum intensity projection, further supported this interpretation (Figure 4b). The most parsimonious interpretation of these data is that filipin signals reveal accumulation of free cholesterol around intracellular parasites that happens early in infection and depending on parasite load sequesters significant amounts.

### 3.4 Analysis of cholesterol distribution with fluorophore‐labeled probes

In order to gain more insight into cholesterol trafficking to the PV, we conducted in vitro infections of cells in which the cholesterol pool was spiked with fluorescent cholesterol probes. For this purpose, we used two fluorophore‐labeled cholesterols, NBD‐ and TopFluor (BODIPY) cholesterol, which are widely used for the study of cholesterol trafficking and distribution in living cells (Maxfield & Wustner, 2012).

Macrophages were preincubated with NBD‐ or TopFluor‐cholesterol and then infected with *L*. *mexicana* amastigotes and fixed at different times postinfection. Figure 5a shows that 72 hr postinfection, both fluorescent cholesterol forms were present in the PV but, surprisingly, did not form a halo around the parasites rather became incorporated into parasite membrane compartments. Double cholesterol staining using filipin and NBD‐cholesterol indicated that the cholesterol halo was already present 1 hr postinfection and that fluorophore‐labeled cholesterol did indeed not contribute at a detectable level to its formation (Figure 5b). Moreover, the presence of NBD‐ and TopFluor‐cholesterol did not interfere with the course of the infection (Figure S7A and S7B, respectively). Thus, these results demonstrate that cholesterol is delivered by two distinct pathways and is differentially accumulated by the parasites suggesting cholesterol source specificity for the generation of the halo.

As carried out for filipin‐stainable cholesterol, we quantified the amount of NBD‐cholesterol taken up by the parasites during the infection. We observed that about 10% of total NBD‐cholesterol was retained by a single parasite, whereas parasites in highly infected macrophages sequestered 37% of this fluorescent cholesterol form (Figure 5c). As demonstrated for filipin staining, we identified that the amount of NBD‐cholesterol associated with the parasites is a function of the number of parasites internalized by the host cell (Figure 5c), showing a similar trend over all time points (data not shown). Quantification of TopFluor‐cholesterol was not possible, because lipid droplets labeled by the TopFluor‐dye are localized too close to the parasites (observation reminiscent of the study of Lecoeur, Giraud, Prevost, Milon, & Lang, 2013, where dendritic cells have been infected with *L*. *amazonensis*) and would falsify the calculation of cholesterol sequestration (Figure S7B). Incorporation of NBD‐cholesterol by *L*. *mexicana* expressing a DsRed transgene 16 hr after infection has also been confirmed by a 3D visualization of a series of images using surface reconstruction (Figure S8A). The cross‐sectional profile of a *z*‐stack evidenced an enhanced DsRed‐NBD colocalization within the parasite and the absence of the NBD‐cholesterol around the parasites (Figure S8B).

### 3.5 Effect of infection on transcription of genes regulating lipid biosynthesis and metabolism

Because internalized parasites are assumed to reside in the “food chain” of the host cell itself and are therefore able to sequester at least a part of the fatty acids that have been proposed to fuel their energy metabolism (Coombs, Craft, & Hart, 1982; Ginger, 2006; Hart & Coombs, 1982), we aimed to further investigate the consequences of this adaptive strategy on the lipid metabolism of infected macrophages. It is commonly known that several transcription factors are activated either by the presence (PPAR‐γ, LXR‐α; Desvergne & Wahli, 1999; Edwards, Kennedy, & Mak, 2002; Tontonoz & Spiegelman, 2008) or absence (sterol regulatory element‐binding protein SREBP; Ikonen, 2008) of particular lipids. Thus, we looked at the possible effects of *Leishmania* infection on host cell lipid homeostasis by analyzing the relative change in the mRNA of target genes of these transcription factors.

**Figure 6:**
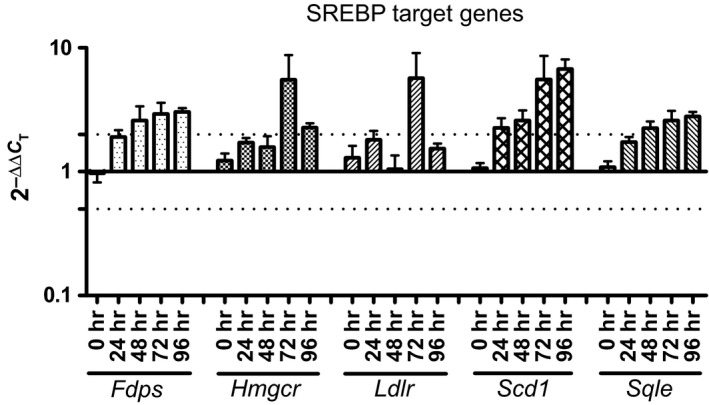
Genes involved in cholesterol synthesis are upregulated upon *Leishmania mexicana* amastigotes infection. Relative mRNA level changes between infected and uninfected bone marrow‐derived macrophages of genes regulated by SREBP. A value of 1 corresponds to no change in gene expression. Dotted lines show either twofold up‐ or downregulation. *Fdps*, farnesyl diphosphate synthase; *Hmgcr*, 3‐hydroxy‐3‐methylglutaryl‐CoA reductase; *Ldlr*, low‐density lipoprotein receptor; *Scd1*, stearoyl‐Coenzyme A desaturase 1, *Sqle*: squalene epoxidase

We found that genes regulated by PPAR‐γ and LXR‐α are essentially not affected by infection (data not shown). In contrast, genes involved in cholesterol synthesis (*Fdps*,* Hmgcr*,* Scd1*, and *Sqle*) under the transcriptional control of SREBP were upregulated 24–48 hr postinfection (Figure 6). The LDL receptor (*Ldlr*), also regulated by SREBP, and involved in LDL uptake, was not consistently upregulated throughout the course of an infection. However, we did observe increased levels of *Ldlr* mRNA at 24 and 96 hr postinfection and at the 72 hr time point, the expression of this gene was increased more than twofold (Figure 6).

## 4 Discussion

In the present work, we investigated the fate of cholesterol in macrophages infected with *Leishmania* parasites and observed a cholesterol redistribution of cellular cholesterol. Based on the findings, we propose increased retention of serum‐derived cholesterol in the PV during the leishmanial infection as a consequence or cause of reduced levels of NPC1, a protein implicated in cholesterol efflux from late endosomes and lysosomes. Retained cholesterol concentrates around parasites forming a kind of coat whose function has yet to be elucidated. Moreover, parasites incorporate host cell membraneous cholesterol. This two‐way cholesterol sequestration can affect significant proportions that become registered by the host cell's cholesterol homeostasis mechanisms which include triggering of SREBP‐mediated transcription of key cholesterol biosynthesis genes.

Cholesterol is a major constituent of eukaryotic membranes and is implicated in several cellular processes such as membrane organization and dynamics, signal transduction, and molecular sorting. It is known that serum levels correlate with distinct occurrence of disease. For instance, hypercholesterolemia protects from, whereas hypocholesterolemia promotes, infection of macrophages by *L*. *donovani* (Ghosh et al., 2012). Recently, it has been documented that low serum cholesterol in mice with visceral leishmaniasis is associated with downregulation of miR‐122 through the degradation of its processor, Dicer1, by the metalloprotease gp63, which inhibits cholesterol biosynthesis in infected mouse liver (Ghosh et al., 2013). In addition, lipoprotein lipase gene polymorphism is associated with high levels of triglycerides and very low‐density lipoproteins (VLDL) and low levels of high‐density lipoprotein (HDL), which favor the development of visceral leishmaniasis (Carvalho et al., 2014).

At the cellular level, cholesterol‐enriched membrane microdomains termed lipid rafts seem essential in *Leishmania* uptake and infection (Majumder et al., 2012; Pucadyil et al., 2004). Cholesterol depletion from the plasma membrane noted after *Leishmania* infection has been proposed to represent an immune evasion strategy used by the intracellular parasites. Lipid rafts‐dependent processes such as MHC–antigen complex formation on the surface of antigen‐presenting cells which is required for a productive T cell response (Anderson, Hiltbold, & Roche, 2000; Burack, Lee, Holdorf, Dustin, & Shaw, 2002) and the function of raft‐associated membrane‐located receptors, such as IFN receptors (Rub et al., 2009; Sen et al., 2011), would thereby be impaired and intracellular parasite survival would be more likely.

Trypanasomatids including *Leishmania* amastigotes cannot synthesize cholesterol de novo (Roberts et al., 2003). Cholesterol detected in the parasites must be obtained from their host cell or environment. It is of note that in lesion derived amastigotes, the content of cholesterol related to the total amount of free sterols increases (Tetley, Coombs, & Vickerman, 1986) raising from 7% to 39% when compared to promastigotes (Berman, Goad, Beach, & Holz, 1986; Ginger, Chance, Sadler, & Goad, 2001). Recently, it has been demonstrated that *Leishmania* parasites are able to acquire significant amounts of cholesterol from LDL particle endocytosis (Andrade‐Neto et al., 2011; De Cicco et al., 2012). This strategy has also been illustrated for other protozoan parasites such as *Toxoplasma gondii*,* Trypanosoma cruzi*, and *Cryptosporidium parvum*, which indicate that cholesterol is widely important for the intracellular survival and replication of pathogens (Coppens, Sinai, & Joiner, 2000; Ehrenman, Wanyiri, Bhat, Ward, & Coppens, 2013; Johndrow et al., 2014; Pereira et al., 2011; Portugal et al., 2008). Our experiments using LDL containing ^3^H‐labeled cholesteryl linoleate suggest that *Leishmania* amastigote‐infected cells do not primarily increase the overall cellular uptake of cholesterol, but rather increased retention of cholesterol in the parasite‐containing subcellular fraction is observed when compared to a similarly prepared fraction containing latex beads. The absence, exclusion, or inhibition of proteins that normally regulate cholesterol efflux from such compartments as shown here for NPC‐1 offers a parsimonious explanation for this observation. This differs substantially from the hypothesis (Rabhi et al., 2012) that *Leishmania* might induce accumulation of cellular cholesterol by downregulating expression of members of the lipid efflux machinery, such as the ATP‐binding cassette transporter A1 (ABCA1), as illustrated for *L. major*‐infected cells, which we could not confirm (D. Paape & T. Aebischer, unpubl. data).

Although the aforementioned studies provide teleological support for a beneficial role of sequestration of serum and plasma membrane cholesterol during *Leishmania* infection in order to protect the parasites from the host cell's antileishmanial activity, the chronology of redistribution and accumulation of cholesterol after infection has not been investigated. In *L. major*‐infected macrophages, it has been reported that cholesterol is localized in the cytosol as free cholesterol and/or esterified with fatty acids and stored in lipid droplets in close proximity to PV, suggesting that lipid droplets act as energy repositories for internalized amastigotes (Rabhi et al., 2012). The latter mechanism has also been proposed for dendritic cells infected with *L*. *amazonensis* (Lecoeur et al., 2013).

With a focus on the parasite, our work suggests that *Leishmania* infection leads to the sequestration and accumulation of filipin‐stainable free cholesterol around the parasites forming a coat which is already detectable in the early phases of the infection. This phenotype appears to be common since it was observed with both, New World (*L*. *mexicana*) and Old World (*L. major*) species, despite their significantly different PVs. In infections with *Toxoplasma gondii* and *Cryptosporidium parvum*, LDL‐derived cholesterol has been proposed to become localized in parasite membranes, but filipin staining suggested a surface accumulation of free cholesterol also in these cases (Coppens et al., 2000; Ehrenman et al., 2013). Because analyses with NBD‐ and TopFluor‐cholesterol probes, which principally become included in the cytoplasma and plasma membrane lipid rafts, respectively (McIntosh et al., 2008), are trafficked to the PV and incorporated into parasites, but are apparently not accumulating around the parasite, we suggest that the halo is predominantly constituted by the part of exogenous likely serum‐derived cholesterol that is trafficked directly into the PVs. Nevertheless, further analyses are required to confirm this hypothesis.

The semiquantitative analysis of cholesterol sequestration revealed a general correlation of parasite number per cell and extent of sequestration. A finding that is an important addition to previously published works. Only at higher parasite loads do ~25% of both host cell total filipin‐stainable free cholesterol and total NBD‐cholesterol become associated with parasites with maximal values of 50% observable. These are values in the ranges reported for *L. major* that in experiments with an MOI of 10 diminished the cholesterol content in macrophages derived from BALB/c mice by about one‐third 72 hr after infection and this correlated with altered CD40 signalosome composition and function (Rub et al., 2009). Similarly, in macrophages derived from C57BL/6 mice and infected with *L*. *donovani* at an MOI of 10, the total host cell membrane cholesterol became decreased by almost 50% 48 hr postinfection (Ghosh et al., 2012). The same parasite species used again at an MOI of 10 reduced plasma cell membrane cholesterol reportedly by two thirds in RAW 264.7 macrophages, 8 hr postinfection. This was accompanied by downregulation of the IFN‐γ receptor signaling in these host cells (Sen et al., 2011). Thus, while cholesterol halo formation that covers the parasite is fast and in its extent per parasite most pronounced at low parasite load, cholesterol sequestration from the host cells point of view becomes sizable and functionally relevant only at higher parasite numbers. Therefore, we like to speculate that halo formation serves a different purpose that is not yet known.

The obvious consequence of cholesterol retention and deprivation reaching functionally relevant levels would be the attempt of the host cell to counteract this trend and to restore the initial cholesterol levels. As reported in our work with *L*. *mexicana*, upregulation of the expression of genes related to the lipid metabolism and cholesterol biosynthesis has been also shown in other studies conducted with *L*. *major* and *L. amazonensis* in macrophages of BALB/c mice (Osorio y Fortea et al., 2009; Rabhi et al., 2012). However, whether this biological response is universal needs further studies, since, for example, in dendritic leukocytes *L. amazonensis* did not influence (Lecoeur et al., 2013), whereas in macrophages derived from human lung lymphoblasts *L. viannia braziliensis* even lead to downregulation of the expression of genes belonging to steroid and sterol biosynthetic processes (Ovalle‐Bracho, Franco‐Munoz, Londono‐Barbosa, Restrepo‐Montoya, & Clavijo‐Ramirez, 2015).

In summary, our study shows a novel dynamic cholesterol sequestration pattern by *Leishmania* spp. in the parasitophorous vacuole of host cell macrophages and identifies changes to trafficking of at least two pools of cellular cholesterol as its mechanistic basis. Quantitative analyses revealed that cholesterol sequestration is dependent on the number of parasites per cell, but is time independent. This finding suggests that *Leishmania* need to reach a relatively high intracellular number to affect the host cell's cholesterol homeostasis to a degree that impairs its immune signaling functions. In contrast, the amount of cholesterol sequestered per parasite is most pronounced at low number of intracellular amastigotes. This might indicate a response to a yet to be elucidated selection pressure encountered during evolution.

## Conflict of Interest

None declared.
