# Supplementary figures and images for: Changes to cholesterol trafficking in macrophages by Leishmania parasites infection
Source: Microbiologyopen. 2017 Mar 27;6(4):e00469. doi: 10.1002/mbo3.469 (PMC5552908; doi:10.1002/mbo3.469)

24h

48h

72h

96h

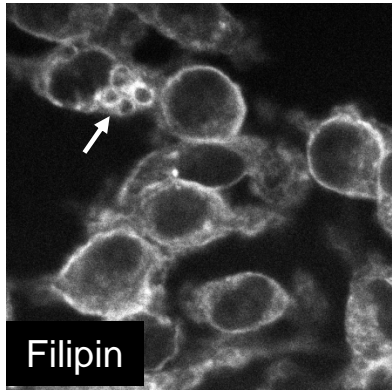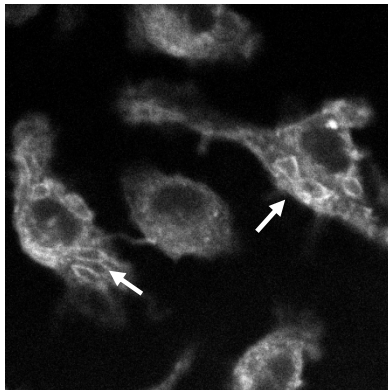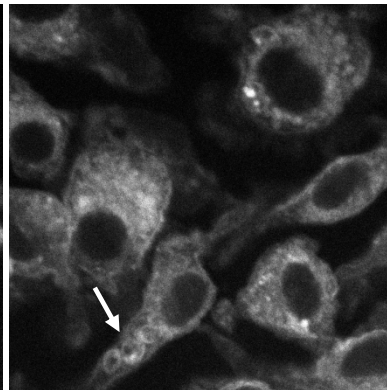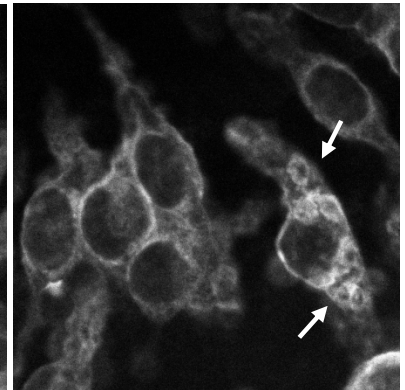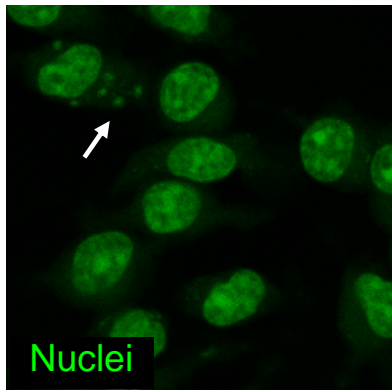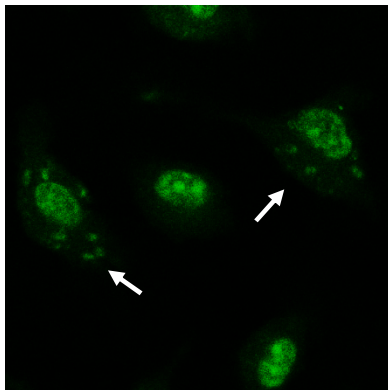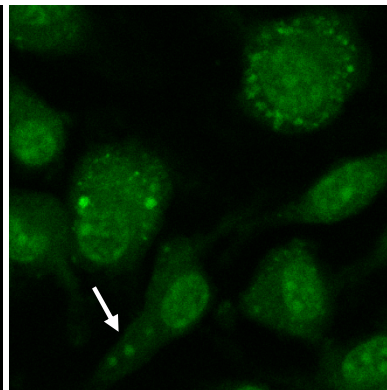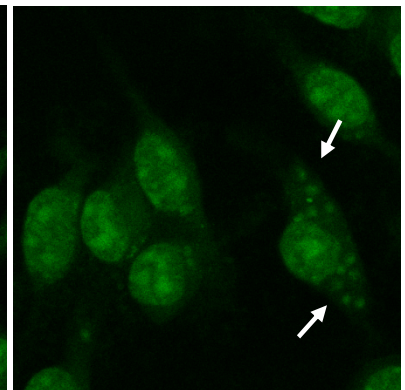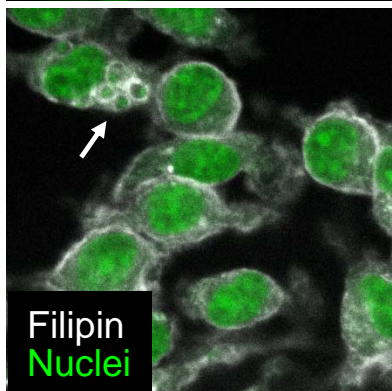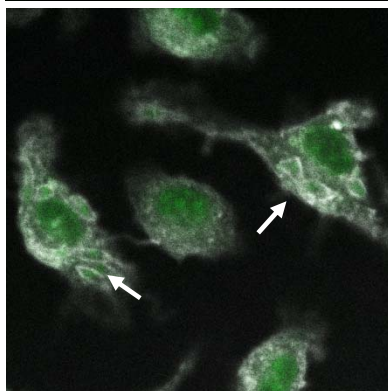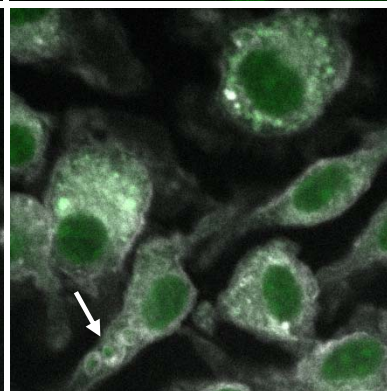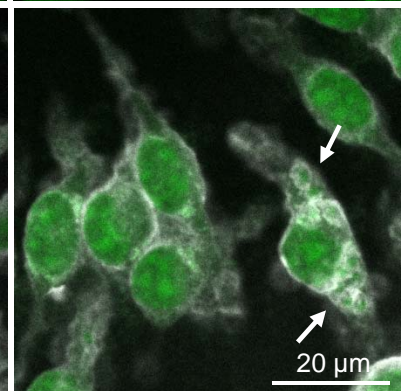

Supplement: Supplementary file 1 [file MBO3-6-na-s001.pdf]

24h

48h

72h

96h

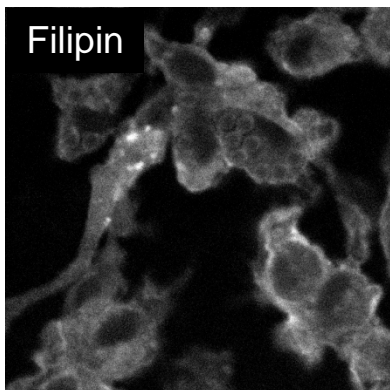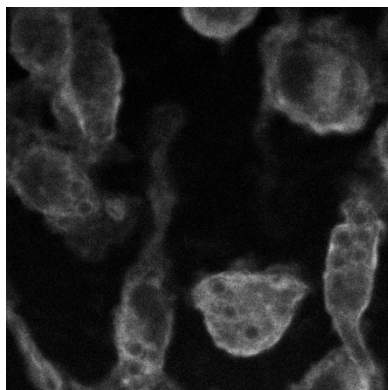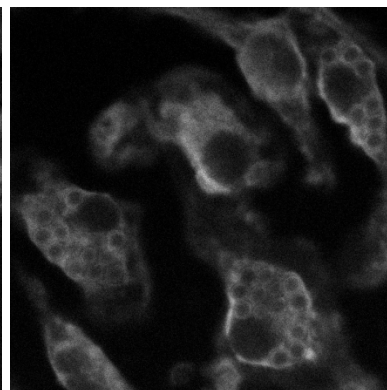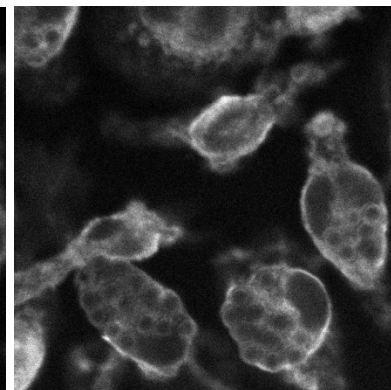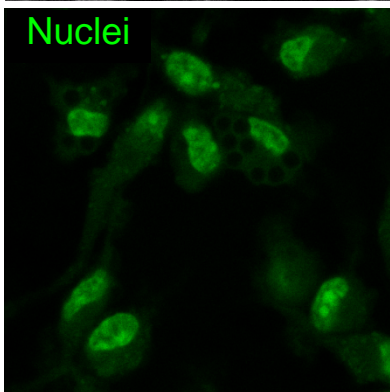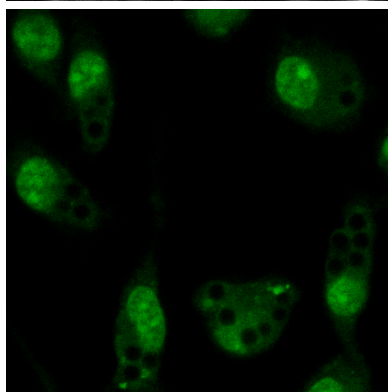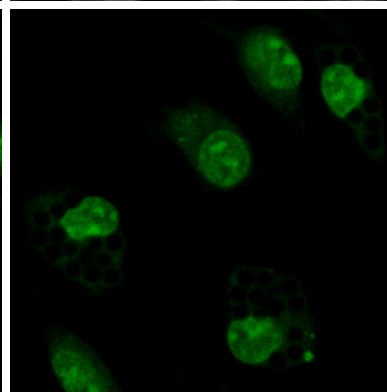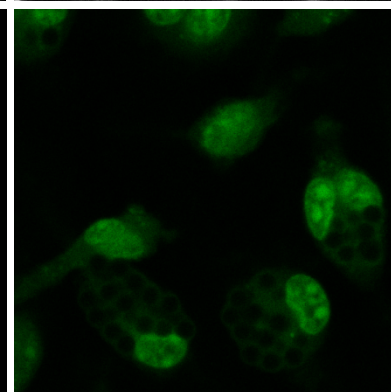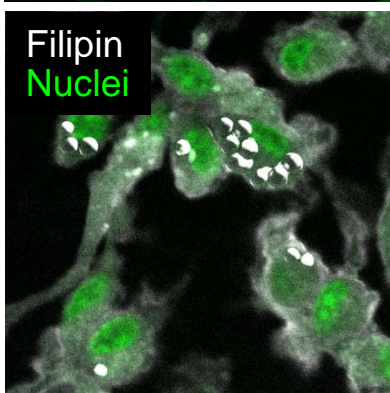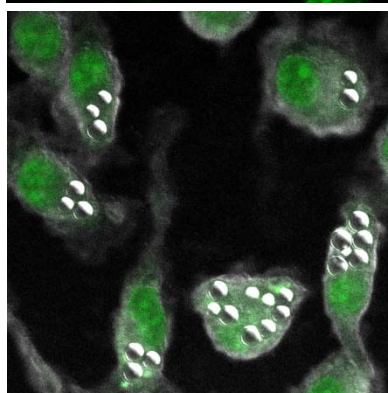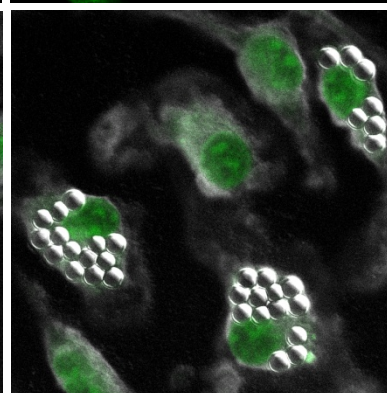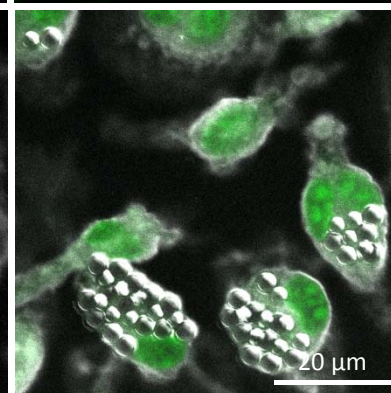

Supplement: Supplementary file 2 [file MBO3-6-na-s002.pdf]

**A**

24 h p.i.

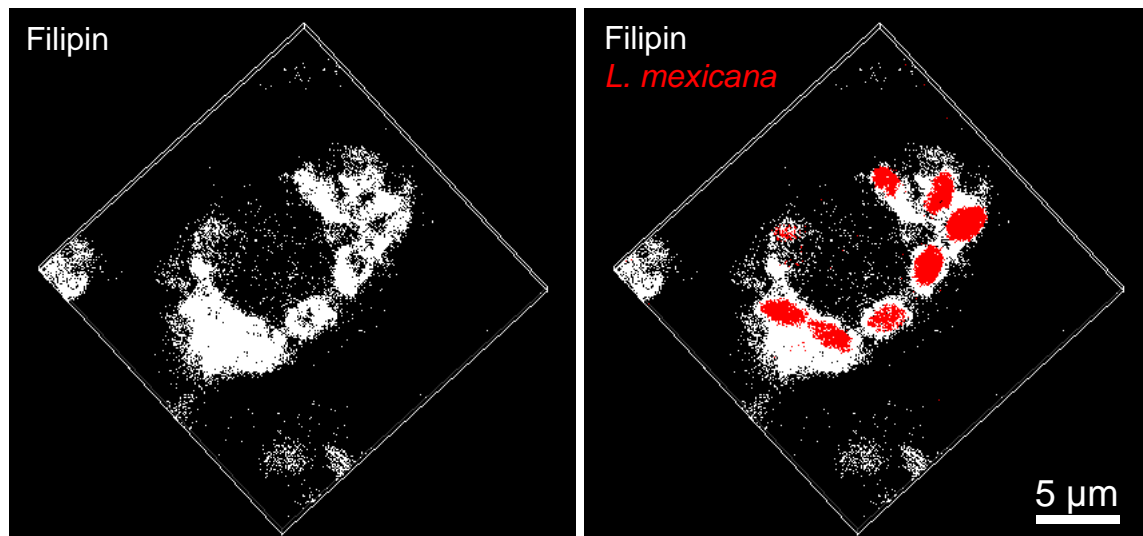**B**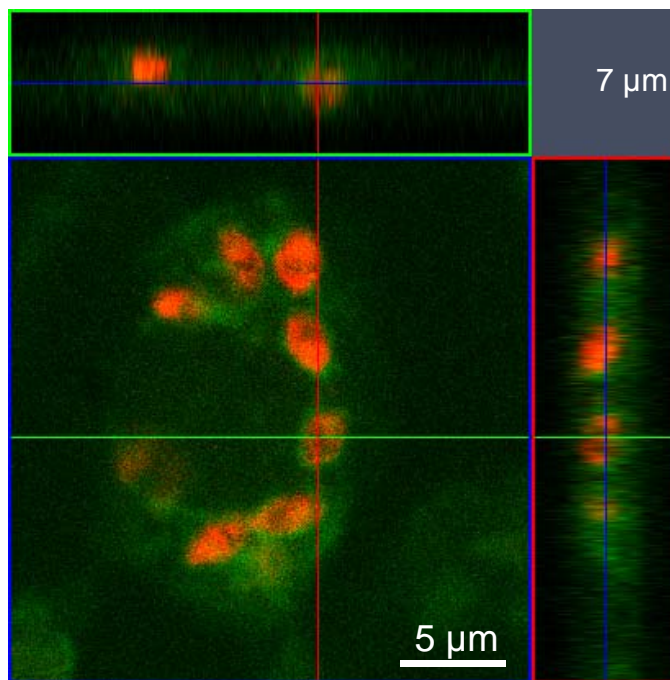

Supplement: Supplementary file 3 [file MBO3-6-na-s003.pdf]

**A**

48 h p.i.

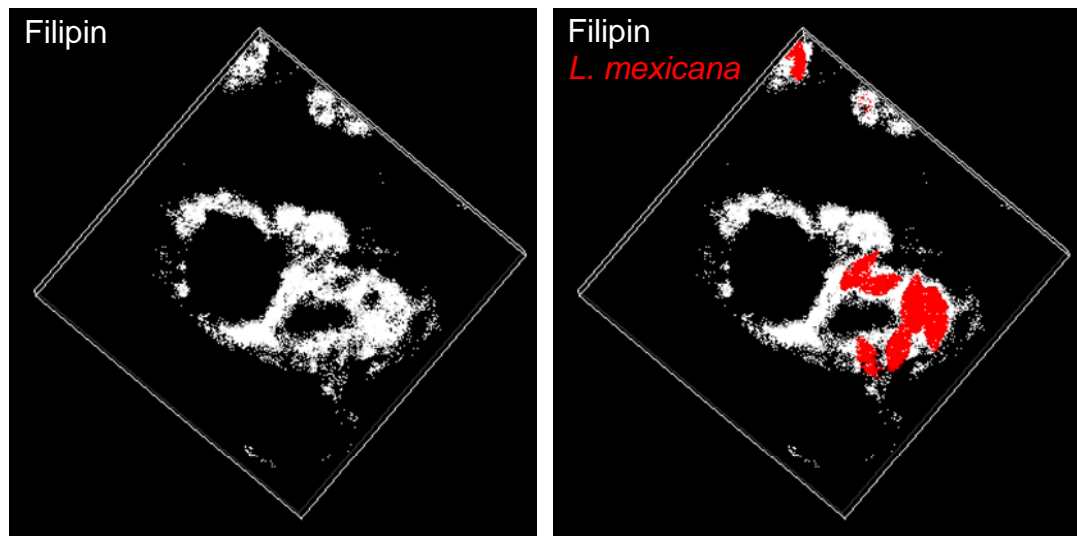**B**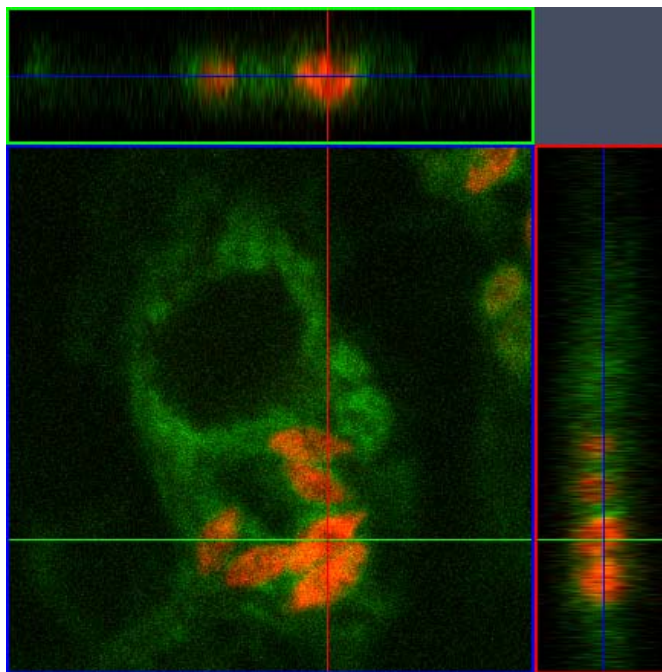

Supplement: Supplementary file 4 [file MBO3-6-na-s004.pdf]

**A**

72 h p.i.

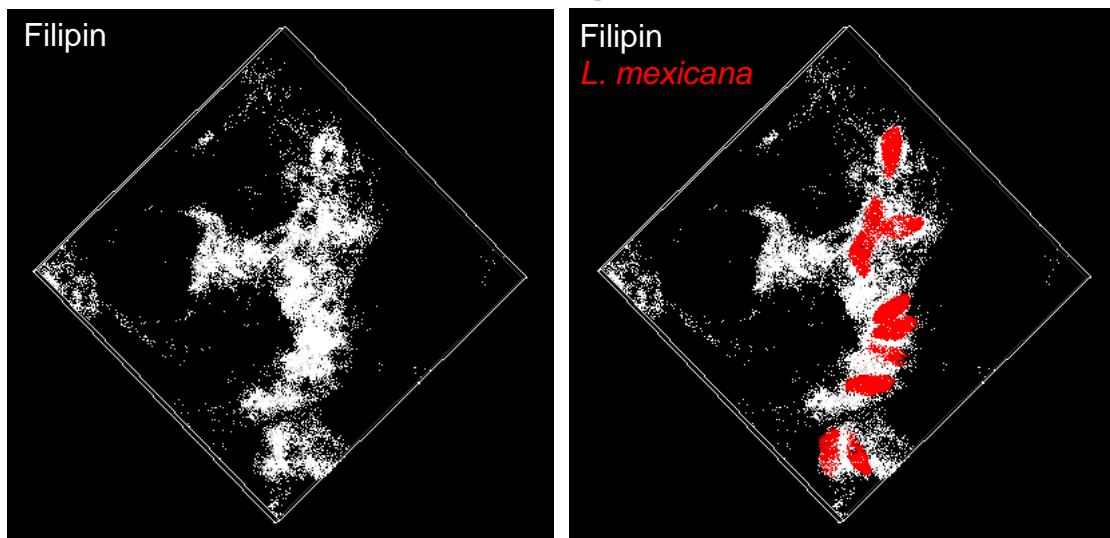**B**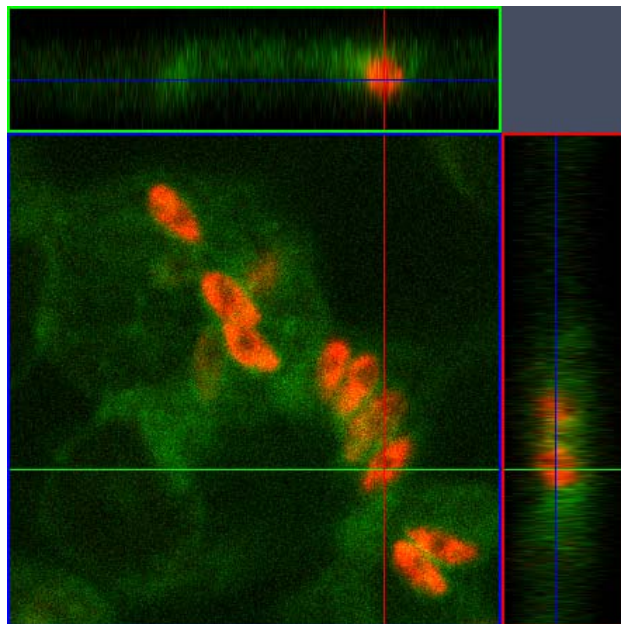

Supplement: Supplementary file 5 [file MBO3-6-na-s005.pdf]

**A**

96 h p.i.

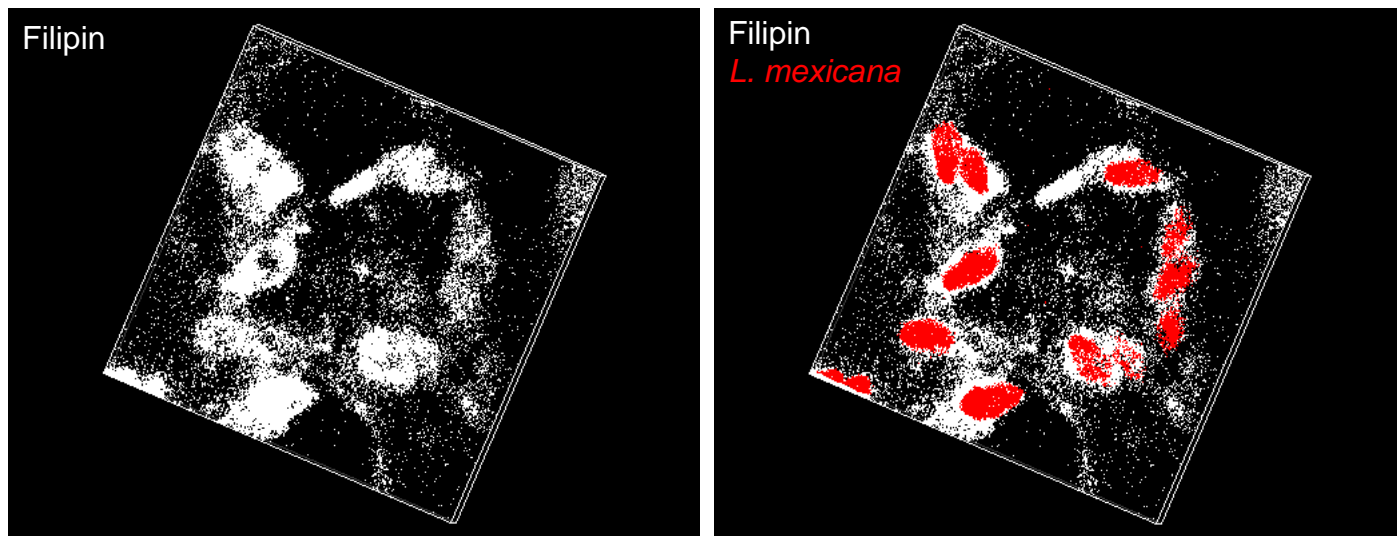**B**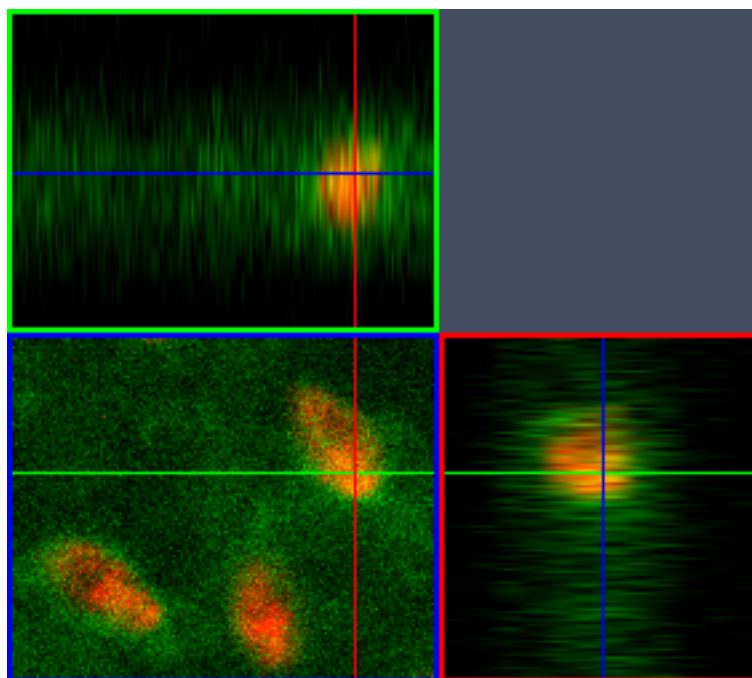

Supplement: Supplementary file 6 [file MBO3-6-na-s006.pdf]

**A**

0 h

4 h

20 h

48 h

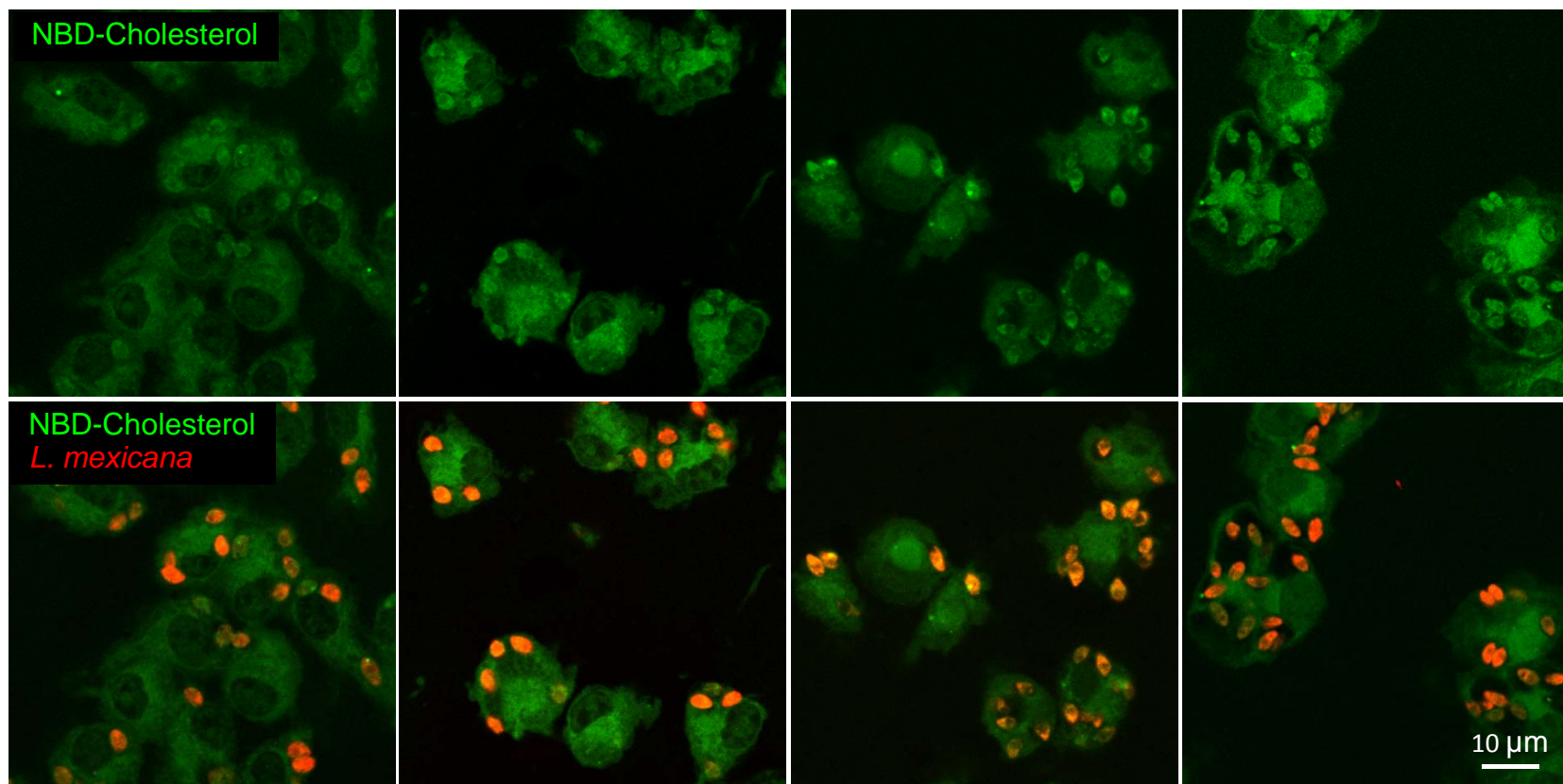**B**

0 h

4 h

20 h

48 h

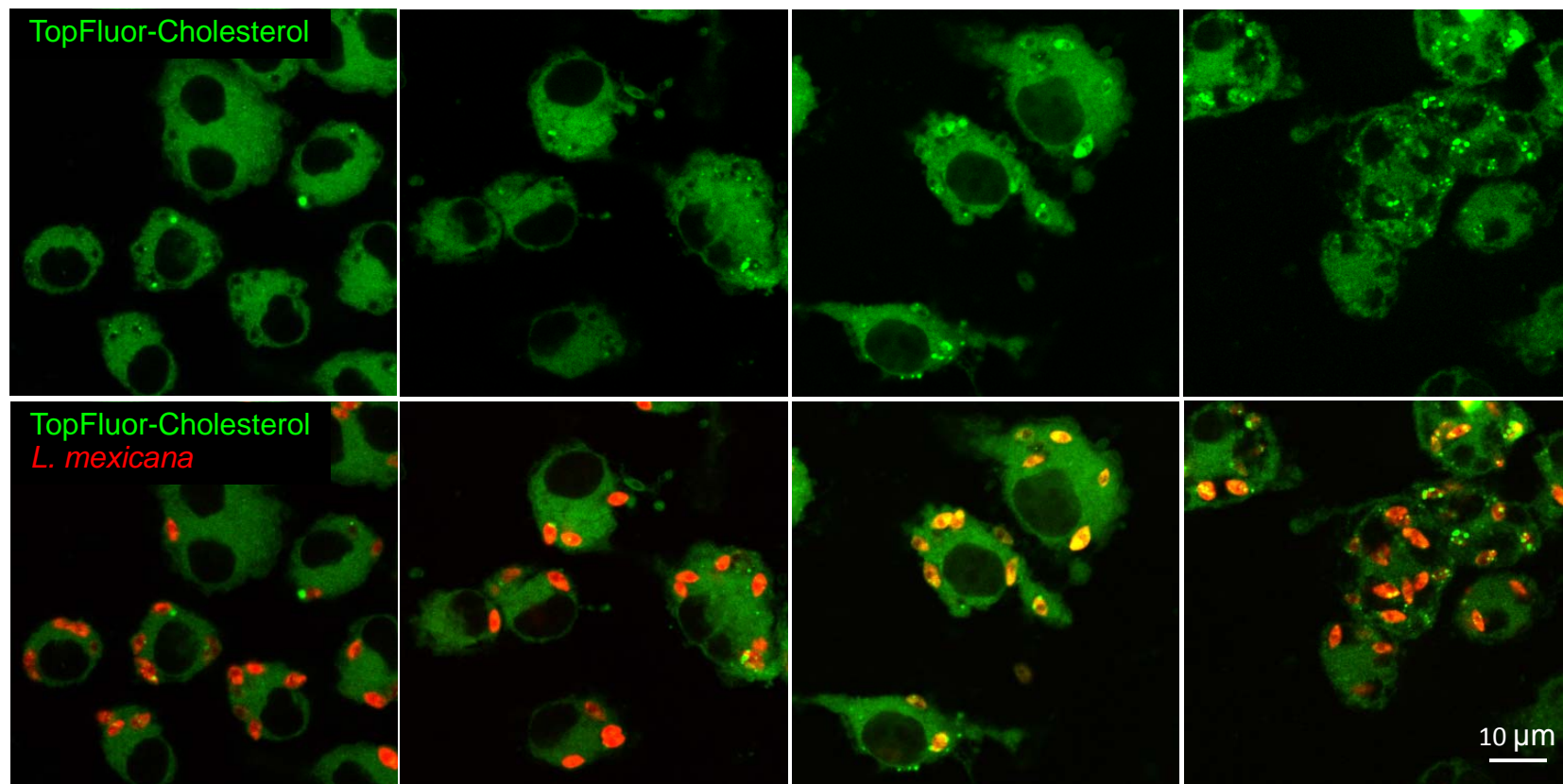

Supplement: Supplementary file 7 [file MBO3-6-na-s007.pdf]

72 h p.i.

**A**

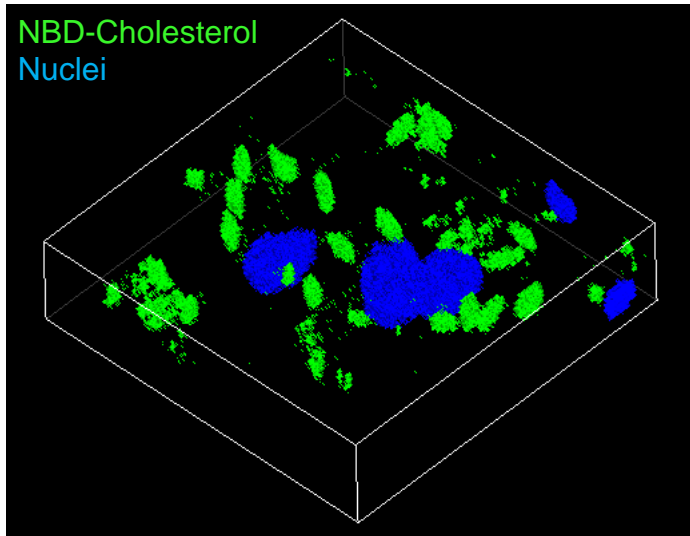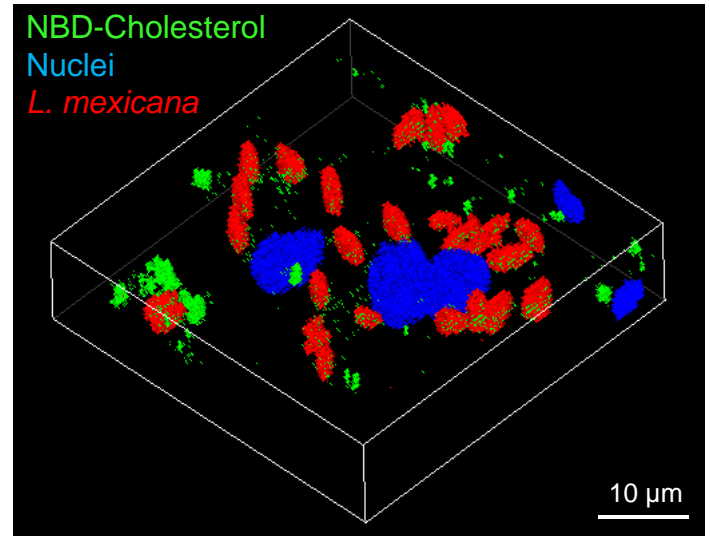

**B**

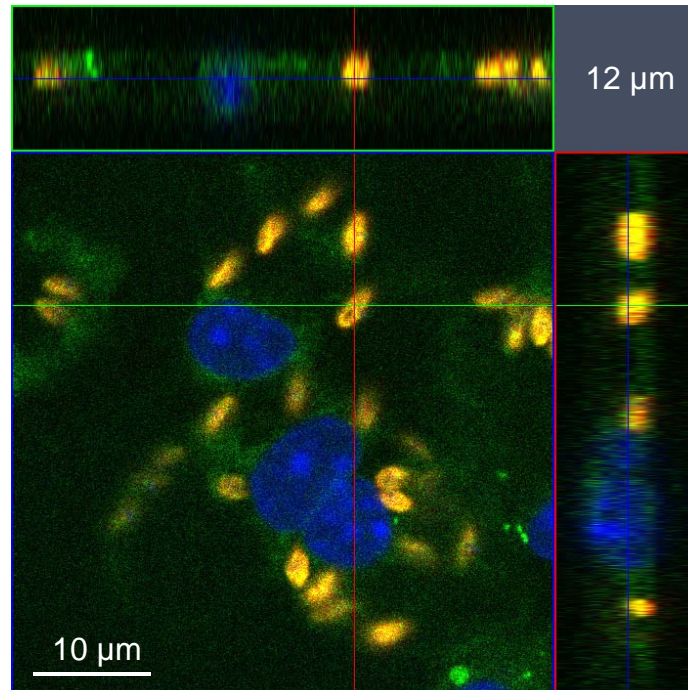

Supplement: Supplementary file 8 [file MBO3-6-na-s008.pdf]
